# Factors Associated with Carbohydrate Counting Adherence in Adults with Type 1 Diabetes Mellitus in Brazil

**DOI:** 10.3390/nu16213594

**Published:** 2024-10-23

**Authors:** Gabriela Correia Uliana, Sarah Emili Cruz da Silva, Manuela Maria de Lima Carvalhal, Carla Cristina Paiva Paracampo, Daniela Lopes Gomes

**Affiliations:** 1 Postgraduate Program in Neurosciences and Behavior, Nucleus of Behavior Theory Research, Federal University of Pará, Belém 66075-110, Brazil; gabriela.ulianafh@gmail.com (G.C.U.); cparacampo@gmail.com (C.C.P.P.); 2 Faculty of Nutrition, Federal University of Pará, Belém 66075-110, Brazil; sarahemili68@gmail.com; 3 Social Service of Commerce, 40 hour road 110, Ananindeua 67120-370, Brazil; manuela.carvalhall@gmail.com

**Keywords:** type 1 diabetes mellitus, carbohydrate counting, glycemic control

## Abstract

**Background:** Carbohydrate Counting (CC) is important in managing the treatment of Type 1 Diabetes Mellitus (T1DM). This study aimed to evaluate the factors associated with adherence to CC in adults with T1DM in Brazil. **Methods:** A cross-sectional study was conducted through an online questionnaire. Information was collected on sociodemographic, economic, clinical, and anthropometric factors; knowledge of the CC strategy; the acquisition of supplies; the perception of mathematical skills as a hurdle in adhering to CC; and follow-up with healthcare professionals. Pearson’s chi-squared or Fisher’s exact test was applied (*p* < 0.05). **Results:** Of the 173 participants, 72.8% practiced CC. Practicing CC was associated with having an income higher than three minimum wage equivalents (*p* = 0.023), and not practicing CC due to the lack of supplies for glucose monitoring was associated with having practiced CC at some point but is currently not practicing (*p* < 0.001). Not practicing the necessary calculations for CC was associated with “knowing how to do CC but had never done it” and “had done CC at some point but currently not practicing” (*p* < 0.001). Stopping or having stopped practicing CC due to insufficient materials for glucose monitoring was associated with having practiced CC for a period but is not currently doing so (*p* < 0.001). Following up with healthcare professionals (*p* < 0.001) and receiving encouragement from the endocrinologist (*p* < 0.001) and nutritionist (*p* = 0.047) were associated with adherence to CC. **Conclusions:** Having a better financial status, performing the mathematical calculations required for CC, having access to supplies for glucose monitoring, and receiving specialized professional follow-up were factors associated with adherence to CC in Brazil.

## 1. Introduction

Type 1 Diabetes Mellitus (T1DM) is an autoimmune condition that leads to the destruction of β-cells, resulting in an absolute insulin deficiency and, consequently, persistent hyperglycemia [1,2]. For this reason, adherence to treatment is essential to maintain the patient’s glycemic levels as close as possible to the target range in order to prevent future complications [3,4].

Carbohydrates (CHO) are the macronutrient that has the most impact on glycemia, as they are fully converted into glucose in the body. Therefore, managing carbohydrate intake helps with glycemic control, allowing for a better prognosis [1,3,5,6]. In this context, Carbohydrate Counting (CC) is an important tool in the treatment of T1DM since the amount of carbohydrates consumed in a meal serves as the parameter for calculating the bolus insulin dose to be administered [1,3].

CC should be implemented within the context of a healthy diet, which is one of the fundamental pillars of treatment as it is directly related to positive outcomes in the other pillars, which include intensive insulin therapy (basal-bolus regimen), regular glucose monitoring, and regular physical exercise [7].

In a study by Deng et al. [8] that examined the association between practicing CC, along with other self-management behaviors, and maintaining adequate glycated hemoglobin (HbA1c) levels in patients with T1DM, it was observed that participants who practiced CC were more likely to have adequate HbA1c levels. AlBabtain et al. [9], in their integrative review, observed similar results, concluding that CC contributed to improved glycemic control.

It is noteworthy that, although CC is a highly effective and advantageous strategy for glycemic control in T1DM patients, it is also considered challenging and complex. It requires individuals to perform a series of behaviors during meals, such as preprandial and postprandial glucose monitoring, calculating the amount of carbohydrates consumed during meals, and adjusting the insulin dose according to preprandial glycemia and the measured carbohydrate intake [7]. Moreover, performing these calculations requires the use of scales, calculators, food measuring tools, nutrition labels, smartphone apps, nutritional tables, and food diaries [1,7]. Therefore, for CC to be practiced accurately, it is essential that patients have access to various equipment and tools that facilitate adherence to this strategy.

Additionally, according to Yari et al. [10] and AlBabtain et al. [9], there are differences in the variety of approaches used for CC, including variations in the tools and methods employed, as well as the level of mastery of the subject by both patients and healthcare professionals. Furthermore, according to Ramalho et al. [11], the lack of demographic characterization, including age, gender, health conditions, and financial status, hampers a clear and comprehensive understanding of the socioeconomic and demographic aspects that could either facilitate or hinder adherence to CC, limiting the allocation of resources toward the practice of CC.

Thus, although research has already demonstrated the benefits of CC, there is still a scarcity of studies analyzing the environmental and sociodemographic variables that may influence adherence to this strategy. It is hypothesized that demographic and economic factors, mathematical knowledge, CC knowledge, clinical data, and access to healthcare professionals are associated with adherence to CC. Thus, the present study aimed to test whether there is an association between environmental and sociodemographic variables and adherence to CC in adults with T1DM in Brazil.

## 2. Materials and Methods

### 2.1. Study Type and Location

This was a cross-sectional, descriptive, and analytical study conducted from November 2021 to June 2022. The research was conducted online, with dissemination, through social media platforms (WhatsApp^®^, Facebook^®^, and Instagram^®^), of an outreach project on nutrition and T1DM, targeting, preferably, individuals who self-identified as having T1DM on social media.

### 2.2. Participants

The participants in this study were the same as those in the study by Uliana et al. [12]. A convenience sample was taken from adults diagnosed with T1DM in Brazil. The inclusion criteria were as follows: individuals diagnosed with T1DM, aged between 18 and 59 years (adult age group), of both sexes; familiar with the Carbohydrate Counting (CC) strategy; and those who agreed to the Informed Consent Form (ICF) by selecting the option “I have read the ICF and AGREE to participate in the study”. The exclusion criteria included being legal guardians of minors with diabetes, children/adolescents with T1DM, individuals with types of diabetes other than T1DM (T2DM, gestational, latent autoimmune diabetes in adults, maturity-onset diabetes of the young, etc.), individuals with diabetes and unable to specify the type; individuals under 18 or over 59 years of age; those unaware of what CC is; and those who did not agree to participate in the study by selecting the option “I do not agree to participate in the study” after reading the ICF. Data from individuals who did not complete the survey were excluded.

### 2.3. Instrument

A structured questionnaire, based on the preliminary study by Uliana et al. [13], was used which underwent content and appearance validation processes [14,15]. Initially, content validation was conducted, followed by appearance validation through the evaluation of three expert judges: one with a Ph.D. and two with a master’s degree. All were nutritionists working in Brazil and had qualifications in diabetes education from the Brazilian Diabetes Society/Juvenile Diabetes Association/International Diabetes Federation.

As presented in Uliana et al. [12], the questionnaire was developed on the Google Forms^®^ platform (https://workspace.google.com/intl/pt-BR/lp/forms/?utm_source=google&utm_medium=cpc&utm_campaign=latam-BR-all-pt-dr-bkws-all-all-trial-e-dr-1707806-LUAC0011908&utm_content=text-ad-none-any-DEV_c-CRE_666246535618-ADGP_Hybrid%20%7C%20BKWS%20-%20EXA%20%7C%20Txt-Forms-KWID_43700057676889044-kwd-10647024857&utm_term=KW_google%20forms-ST_google%20forms&gad_source=1&gclid=Cj0KCQjwmt24BhDPARIsAJFYKk1QmdVqB5OJ06J3qhXEbywdfLqC1lo5n35yIlQIoLIkQmz7Kg4g4McaAkG4EALw_wcB&gclsrc=aw.ds, accessed on 20 October 2024), in the format of an opinion survey, in accordance with Resolution 510 of 7 April 2016 [16]. The questions were divided into the following sections.

1. Knowledge of CC: questions regarding knowledge on how to practice CC, when CC is practiced, tools used to measure the amount of carbohydrates in food, whether a kitchen scale is used for CC, and why a kitchen scale is used. It should be noted that the method used in the study was CC in grams.
2. Sociodemographic and economic data: questions related to age, biological gender, region of the country, education level, and family income.
3. Clinical and anthropometric data: questions concerning the time of diagnosis, weight, height, HbA1c test results, and the value of the most recent HbA1c test performed by the participants.
4. Acquisition of supplies: questions about the technology used to administer insulin (insulin pump, pen, syringe, or both pen and syringe), technology used for glucose monitoring (glucometer, Continuous Glucose Monitoring System (CGMS), or Flash system (FGMS), or whether glucose monitoring is not practiced), and whether the participant had ever stopped practicing CC due to a lack of supplies needed for insulin administration or glucose monitoring.
5. Perception of mathematical skills as a hurdle: question about whether difficulty in practicing “mathematical rule of three” calculations affects adherence to CC, with the following response options: very much, a little, it does not affect it, or I don’t know. Participants were also asked if they believe that knowing how to calculate the amount of insulin needed to cover carbohydrate intake affects adherence, and whether knowing how to calculate the amount of insulin needed to correct blood glucose affects adherence, with the response options being very much, a little, it does not affect it, I don’t know, or I’m not familiar with this calculation. Additionally, participants were asked how they usually practice the mathematical calculations for CC (manually, with a calculator, using a smartphone app, manually and with an app, with a calculator and an app, or I do not practice the calculations).
6. Follow-up with healthcare professionals (in the three months prior to the survey): questions on whether follow-up was conducted in person, virtually, both, or not at all; whether the follow-up was conducted through private health insurance, SUS (Brazil’s public health system), both, or privately; and who taught them how to practice CC.

### 2.4. Procedure

The survey link was sent directly to individuals who declared that they have T1DM in their social media status (WhatsApp^®^, Facebook^®^, and Instagram^®^). Upon clicking the link, the participants were first directed to a summary explanation of the study (Informed Consent Form—ICF), which specified that no identification of participants would be required and described the research objectives and methodology in accessible language. After reading the ICF, the participants could choose whether or not to participate. If they clicked the option “I have read the ICF and AGREE to participate in the study”, they were directed to a page where whether they fulfilled the other inclusion criteria was checked.

After answering all the questionnaire questions, the participants were directed to the survey’s closing page, where a link to the “Carbohydrate Counting Manual for People with Diabetes” [7] was made available. This manual provides guidelines for practicing CC and nutritional information about food.

### 2.5. Statistical Analysis

The Statistical Package for Social Science, version 24.0, was used for the statistical analysis. Descriptive results were expressed as absolute frequencies and proportions. To test the association between variables, Pearson’s Chi-Square or Fisher’s exact test was applied, with an adjusted residual analysis, considering a statistical significance level of *p* < 0.05.

### 2.6. Ethical Aspects

The research complied with the legal requirements of Resolutions 466 of 12 December 2012 and 510 of 7 April 2016, published by the National Health Council, which follows the Declaration of Helsinki for studies involving human subjects [16]. The study was approved by the Research Ethics Committee under opinion No. 5.077.488.

## 3. Results

Of the 260 people who responded to the survey, 87 were excluded for not meeting the inclusion criteria. A total of 173 adults with T1DM participated, with an average age of 28.91 ± 7.20 years. The participants’ socioeconomic and demographic characteristics were presented in Uliana et al. [12].

Most of the participants practiced CC (72.8%) and practiced it during the main meals: breakfast (83.8%), lunch (85.5%), and dinner (82.7%). CC was also practiced during morning snacks (57.8%), afternoon snacks (70.5%), and supper (52.0%). Approximately half of the participants used specific CC apps to check the carbohydrate content of foods (50.3%) and used a food scale to practice CC (58.4%), though only for some meals (38.2%). Additionally, most participants reported knowing how to perform mathematical calculations such as subtraction (96.0%), addition (97.7%), and division (94.2%).

In Table 1, it can be observed that being female was associated with practicing CC, while being male was associated with knowing how to practice CC but had never practiced it (*p* = 0.008). Practicing CC was associated with having a family income higher than three minimum wage equivalents (*p* = 0.023). Additionally, believing that the level of education does not influence the lack of adherence to CC was associated with knowing how to practice CC but had never practiced it, and with having practiced CC for a period but is not currently practicing it (*p* = 0.037).

Table 2 shows that using an insulin pump for insulin administration (*p* = 0.001) and using a CGMS or FGMS (*p* = 0.035) were associated with practicing CC.

Additionally, Table 2 indicates an association between not knowing if one had ever stopped practicing CC due to insufficient insulin and knowing how to practice CC but had never practiced it (*p* < 0.001). Stopping or having stopped practicing CC due to insufficient materials for glucose monitoring was associated with having practiced CC for a period but is not currently doing so (*p* < 0.001).

Table 3 shows that manually performing the calculations was associated with practicing CC (*p* < 0.001). However, not performing the necessary mathematical calculations for CC was associated with knowing how to practice CC but had never practiced it, as well as having practiced CC for a period but is not currently practicing it (*p* < 0.001).

Table 4 shows that follow-up with healthcare professionals through health insurance and SUS (*p* < 0.001), having had both in-person and online consultations in the past three months (*p* = 0.010), and receiving encouragement from an endocrinologist (*p* < 0.001) and nutritionist (*p* = 0.047) to practice CC were associated with practicing CC.

An association was found between follow-up with healthcare professionals only through SUS (*p* < 0.001), not having had consultations in the past three months (*p* = 0.010), receiving follow-up from a general practitioner (*p* = 0.005), and not receiving encouragement to practice CC (*p* < 0.001) with knowing how to perform CC but had never practiced it (Table 4).

Follow-up with healthcare professionals only through SUS (*p* < 0.001) and not receiving encouragement to practice CC (*p* < 0.001) were also associated with having practiced CC for a period but is not currently practicing it (Table 4).

## 4. Discussion

This study evaluated the factors associated with adherence to CC in adults with T1DM in Brazil. At the time of data collection, 72.8% of the 173 participants reported using CC in their dietary routine. CC is recognized by the Sociedade Brasileira de Diabetes (SBD) [7] and American Diabetes Association (ADA) [1] as a tool that offers greater autonomy in food choices for people with T1DM and should be considered in treatment, within the context of a healthy diet, to provide stability for other aspects of treatment.

It was observed that being female was associated with practicing CC, while being male was associated with knowing how to practice CC but had never practiced it. No studies were found comparing CC practices between genders; however, it is documented that men are more resistant to seeking healthcare services as self-care is still often considered a feminine practice due to beliefs that it relates to vulnerability and weakness. Additionally, Da Silva et al. [17] noted in their integrative literature review that neglect of prevention, fear of severe diagnosis, and feelings of embarrassment are aspects that contribute to the perpetuation of this scenario.

Although no significant results were observed in relation to age and region of residence, it was observed that the majority of participants aged between 25 and 44 years and that the majority of residents of the southeast region practiced CC. No studies were found that related adherence to CC among different age groups; however, studies such as that of Freire and Ferreira [18] reported that among the factors affecting adherence to DM treatment is the search for information about the disease. The interest in researching information and a greater awareness about the importance of self-care for the adequate treatment of T1DM are aspects that lead adults to be more engaged in the treatment of the disease compared to other age groups.

Furthermore, socioeconomic inequality between Brazilian regions is a factor that exerts a great influence on access to quality healthcare and, consequently, on favorable conditions for adherence to CC since the concentration of income in certain states to the detriment of others influences the coverage of access to Primary Health Care (PHC), availability of services offered, and the number of people that can be reached [19,20]. Therefore, the hypothesis is that living in states with a greater concentration of income and access to healthcare services is related to better conditions for adherence to T1DM treatment.

Practicing CC was associated with having a higher family income while believing that the level of education does not influence non-adherence was associated with not practicing CC. Although the latter association highlights aspects related to individual perception, these data underscore the relevance of this variable in the context of CC. These results are similar to those found in the studies by Reis et al. [19] and Cervantes-Torres and Romero-Blanco [21], where income and education were observed to influence glucose monitoring and adherence to CC.

Income can impact the acquisition of necessary supplies for glucose monitoring and decision-making regarding insulin administration and carbohydrate intake [19], as well as access to specialized healthcare professionals [22].

Regular glucose self-monitoring and insulin administration are required for adherence to CC, and although basic T1DM treatment supplies are provided by the public health system in Brazil, there are no guarantees of regular acquisition, leading some patients to cover a portion of these supplies using personal resources [19,22,23].

Regarding education, the study by Cervantes-Torres and Romero-Blanco [21] found that patients with higher levels of education had better access to information and better glycemic control. This, combined with the results of the present study, supports the hypothesis that financial income and education levels of individuals with T1DM are proportional to their adherence to CC. Educational level can impact the understanding of CC instructions, as practicing CC requires comprehension of mathematical calculations, reading nutritional labels, and managing smartphone applications, among other skills.

This study observed that using more advanced technologies for insulin administration and glucose monitoring was associated with practicing CC. These results align with the ones of the study by Viana et al. [23], which found that not monitoring capillary glucose was associated with not practicing CC. Thus, using technologies for insulin administration and glucose monitoring can contribute to adherence to appropriate T1DM treatment. The study by Cervantes-Torres and Romero-Blanco [21], involving T1DM patients in Spain, compared the clinical data of patients using and not using a Flash Glucose Monitoring System (FGMS). They observed that patients using an FGMS had better adherence to diet and carbohydrate intake management, better medication usage, improved glycemic values, and consequently, a lower risk of developing macrovascular and microvascular complications due to glycemic variability.

In the present study, it was observed that having previously stopped practicing CC due to a lack of sufficient supplies was associated with not currently practicing CC. The study by Reis et al. [19] assessed socioeconomic factors related to the acquisition of supplies for T1DM treatment during the COVID-19 pandemic and found that individuals without regular access to supplies tended to ration them, reusing disposable lancets and reducing the frequency of insulin administration and glucose monitoring. Such practices can result in higher frequencies of hypoglycemia and hyperglycemia. The study by Viana et al. [23] also found that symptoms of stress and anxiety were associated with the irregular receipt of supplies. Together, these findings indicate that irregular access to supplies can impact the motivation for adherence to CC.

The results showing that not performing the necessary mathematical calculations for CC was associated with not currently practicing CC support the findings by Guimarães and Araújo [24], who aimed to investigate the importance of mathematics for the proper management of T1DM. They observed a relationship between glycemic control and the application of mathematical concepts in daily routines through CC and the basal-bolus regimen. These results suggest that a lack of mathematical skills and text interpretation can be obstacles to the appropriate management of T1DM.

It was observed that following up with healthcare professionals through health plans/insurance and SUS, having both in-person and online consultations, and being encouraged to practice CC by endocrinologists and nutritionists were associated with practicing CC. This indicates that healthcare professionals are adhering to the guidelines set by the SBD [25], which recommend that the multidisciplinary healthcare team be composed of qualified professionals with updates to ensure the effectiveness of the implemented therapy to be capable of guiding and providing information on T1DM treatment.

In the review by Damaceno et al. [26], the authors described the current digital tools used in diabetes treatment and observed that patients’ HbA1c levels improved after regular follow-up with healthcare teams compared to using oral medications alone. This demonstrates that professional healthcare follow-up supports treatment adherence.

It should be noted that, although this study included participants from across Brazil, one limitation is that the sample used is not representative, as there is a profile of adults inclined to participate in this type of questionnaire, and the digital format of the questionnaire may hinder participation from individuals without regular internet access. Despite this, the study provides important data on barriers and difficulties that should be considered when conducting diabetes education and providing instructions on practicing CC, which are crucial for managing T1DM. Future studies evaluating CC adherence in different socioeconomic contexts and with the use of various technologies for managing glycemic control in individuals with T1DM may provide further insights into the factors influencing CC. Additionally, intervention studies testing different CC teaching methodologies could help identify strategies that facilitate adherence to CC and contribute to the planning of treatments for patients with T1DM.

## 5. Conclusions

Based on the obtained results, it can be concluded that a better financial status, performing the mathematical calculations required for CC, having access to supplies for glucose monitoring, and receiving specialized professional follow-up were factors associated with adherence to CC among adults with T1DM in Brazil. Therefore, it is important to identify the difficulties in practicing CC so that the teaching of this strategy can be adapted to each patient’s reality, thereby increasing the chances of adherence to this crucial strategy for glycemic control in individuals with T1DM.

## Figures and Tables

**Table 1:** Association between socioeconomic and demographic data and adherence to Carbohydrate Counting in adults with Type 1 Diabetes Mellitus in Brazil, 2022.

	Adherence to CC			
	I Know How to Do It but I’ve Never Done CC	I Have Done It but Currently, I’m Not Practicing It	I Do CC	*p*-Value *
	n (%)	n (%)	n (%)	
Sex				
Male	3 (1.7) ^(+)^	10 (5.8)	14 (8.1) ^(−)^	0.008 ^†^
Female	3 (1.7) ^(−)^	31 (17.9)	112 (64.7) ^(+)^	
Age (years old)				
18–24	1 (0.6)	14 (8.1)	36 (20.8)	0.841
25–44	5 (2.9)	26 (15.0)	85 (49.1)	
45–59	0 (0)	1 (0.6)	5 (2.9)	
Country region				
North	2 (1.2)	3 (1.7)	5 (2.9)	0.063
Northeast	2 (1.2)	12 (6.9)	22 (12.7)	
Midwest	0 (0)	7 (4.0)	32 (18.5)	
Southeast	1 (0.6)	10 (5.8)	41 (23.7)	
South	1 (0.6)	9 (5.2)	26 (15.0)	
Education				
No higher education	3 (1.7)	26 (15.0)	53 (30.6)	0.059
Higher education	3 (1.7)	15 (8.7)	73 (42.2)	
Family income **				
Up to 3 MWs	5 (2.9)	23 (13.3)	49 (28.3) ^(−)^	0.023 ^†^
More than 3 MWs	1 (0.6)	18 (10.4)	77 (44.5) ^(+)^	
Believes level of education influences the lack of adherence to CC				
Very much	3 (1.7)	18 (10.4)	68 (39.3)	0.037 ^†^
A little	0 (0)	13 (7.5)	45 (26.0)	
Does not influence	3 (1.7) ^(+)^	10 (5.8) ^(+)^	12 (6.9) ^(−)^	
Don’t know	0 (0)	0 (0)	1 (0.6)	
Believes financial status influences the lack of adherence to CC				
Very much	2 (1.2)	16 (9.2)	44 (25.4)	0.995
A little	2 (1.2)	12 (6.9)	35 (20.2)	
Does not influence	2 (1.2)	12 (6.9)	43 (24.9)	
Don’t know	0 (0)	1 (0.6)	4 (2.3)	

* Chi-square; ^†^ statistically significant. Residue Analysis: ^(+)^ the number of cases in this cell is significantly larger than what would be expected if the variables were independent; ^(−)^ the number of cases in this cell is significantly smaller than what would be expected if the variables were independent. CC = Carbohydrate Counting; MW, minimum wage equivalent; ** minimum wage = BRL 1100.

**Table 2:** Association between adherence to Carbohydrate Counting and clinical data, insulin administration method, and glucose monitoring in adults with Type 1 Diabetes Mellitus in Brazil, 2022.

	Adherence to CC			
	I Know How to Do It but I’ve Never Done CC	I Have Done It but Currently, I’m Not Practicing It	I Do CC	*p*-Value *
	n (%)	n (%)	n (%)	
BMI classification categories				
Eutrophic	3 (1.7)	24 (13.9)	77 (44.5)	0.278
Malnutrition	0 (0)	4 (2.3)	3 (1.7)	
Overweight	3 (1.7)	13 (7.5)	46 (26.6)	
Time since diagnosis				
Less than 10 years	1 (0.6)	12 (6.9)	36 (20.8)	0.809
More than or equal to 10 years	5 (2.9)	29 (16.8)	90 (52.0)	
Insulin administration method				
Insulin pump	0 (0)	2 (1.2) ^(−)^	49 (28.3) ^(+)^	0.001 ^†^
Pen	4 (2.3)	30 (17.3) ^(+)^	62 (35.8) ^(−)^	
Syringe	0 (0)	3 (1.7)	2 (1.2)	
Both (pen and syringe)	2 (1.2)	6 (3.5)	13 (7.5)	
Stopping or has stopped practicing CC due to insufficient insulin				
Yes, a few times	0 (0)	9 (5.2)	25 (14.5)	0.001 ^†^
Yes, frequently	0 (0)	2 (1.2)	3 (1.7)	
No, I have never	4 (2.3)	29 (16.8)	97 (56.1)	
I don’t know	2 (1.2) ^(+)^	1 (0.6)	1 (0.6) ^(−)^	
Tool for glucose monitoring				
Glucometer	6 (3.5)	32 (18.5)	79 (45.7) ^(−)^	0.035 ^†^
CGMS or FGMS	0 (0)	8 (4.6)	47 (27.2) ^(+)^	
I do not monitor glucose	0 (0)	1 (0.6)	0 (0)	
Stopping or has stopped practicing CC due to insufficient materials for glucose monitoring				
Yes, a few times	2 (1.2)	9 (5.2)	32 (18.5)	0.001 ^†^
Yes, frequently	0 (0)	4 (2.3) ^(+)^	2 (1.2) ^(−)^	
No, I have never	2 (1.2)	25 (14.5)	91 (52.6)	
I don’t know	2 (1.2)	3 (1.7)	1 (0.6)	

* Chi-square; ^†^ statistically significant. Residue Analysis: ^(+)^ the number of cases in this cell is significantly larger than what would be expected if the variables were independent; ^(−)^ the number of cases in this cell is significantly smaller than what would be expected if the variables were independent. CC = Carbohydrate Counting; BMI: Body Mass Index; CGMS = Continuous Glucose Monitoring System; FGMS = Flash Glucose Monitoring System.

**Table 3:** Association between mathematical skills and adherence to Carbohydrate Counting in adults with Type 1 Diabetes Mellitus (Brazil, 2022).

	Adherence to CC			
	I Know How to Do It but I’ve Never Done CC	I Have Done It but Currently, I’m Not Practicing It	I Do CC	*p*-Value *
	n (%)	n (%)	n (%)	
Believes that using the “mathematical rule of three” to calculate the carbohydrate intake from meals influences the lack of adherence to CC				
Very much	2 (1.2)	17 (9.8)	49 (28.3)	0.888
A little	1 (0.6)	11 (6.4)	37 (21.4)	
It does not affect it	3 (1.7)	10 (5.8)	33 (19.1)	
I don’t know	0 (0)	3 (1.7)	7 (4.0)	
Believes that the mathematical formula used to calculate the amount of insulin needed to cover carbohydrate intake influences the lack of adherence to CC				
Very much	3 (1.7)	15 (8.7)	48 (27.7)	0.401
A little	1 (0.6)	16 (9.2)	38 (22.0)	
It does not affect it	1 (0.6)	7 (4.0)	33 (19.1)	
I don’t know	0 (0)	1 (0.6)	5 (2.9)	
I’m not familiar with this calculation	1 (0.6)	2 (1.2)	2 (1.2)	
Believes that the mathematical formula used to calculate the amount of insulin needed to correct blood glucose influences the lack of adherence to CC				
Very much	3 (1.7)	14 (8.1)	46 (26.6)	0.540
A little	1 (0.6)	11 (6.4)	35 (20.2)	
It does not affect it	1 (0.6)	13 (7.5)	38 (22.0)	
I don’t know	0 (0)	2 (1.2)	5 (2.9)	
I’m not familiar with this calculation	1 (0.6)	1 (0.6)	2 (1.2)	
Method usually used to perform the necessary mathematical calculations for CC				
Manually	0 (0)	1 (0.6) ^(−)^	18 (10.4) ^(+)^	0.001 ^†^
With a calculator	1 (0.6)	8 (4.6)	32 (18.5)	
Using a smartphone app	1 (0.6)	7 (4.0)	27 (15.6)	
Manually and with a calculator	0 (0)	6 (3.5)	14 (8.1)	
Manually and with a smartphone app	0 (0)	1 (0.6)	9 (5.2)	
With a calculator and a smartphone app	0 (0)	7 (4.0)	22 (12.7)	
I do not perform the calculations	4 (2.3) ^(+)^	11 (6.4) ^(+)^	4 (2.3) ^(−)^	

* Chi-square. ^†^ statistical significant. Residue Analysis: ^(+)^ the number of cases in this cell is significantly larger than what would be expected if the variables were independent; ^(−)^ the number of cases in this cell is significantly smaller than what would be expected if the variables were independent. CC = Carbohydrate Counting.

**Table 4:** Association between follow-up with health professionals and adherence to Carbohydrate Counting in adults with T1DM in Brazil, 2022.

	Adherence to CC			
	I Know How to Do It but I’ve Never Done CC	I Have Done It but Currently, I’m Not Practicing It	I Do CC	*p*-Value *
	n (%)	n (%)	n (%)	
Coverage for follow-up with health professionals				
Health plan/insurance	0 (0)	11 (6.4)	42 (24.3)	0.001 ^†^
SUS	4 (2.3) ^(+)^	16 (9.2) ^(+)^	18 (10.4) ^(−)^	
Both (health plan/insurance and SUS)	2 (1.2)	1 (0.6) ^(−)^	29 (16.8) ^(+)^	
Private service	0 (0)	13 (7.5)	37 (21.4)	
In the last three months, I had consultations				
In person	2 (1.2)	27 (15.6)	71 (41.0)	0.010 ^†^
Online	0 (0)	1 (0.6)	8 (4.6)	
In person and online	0 (0)	2 (1.2) ^(−)^	27 (15.6) ^(+)^	
I haven’t had any consultations	4 (2.3) ^(+)^	11 (6.4)	20 (11.6) ^(−)^	
Health professional who performed follow-up				
Endocrinologist	6 (3.5)	38 (22.0)	123 (71.1)	0.290
General Practitioner	3 (1.7) ^(+)^	9 (5.2)	12 (6.9) ^(−)^	0.005 ^†^
Nutritionist	1 (0.6)	18 (10.4)	55 (31.8)	0.421
Nurse	0 (0)	4 (2.3)	11 (6.4)	0.729
Psychologist	0 (0)	10 (5.8)	34 (19.7)	0.328
Other	0 (0)	3 (1.7)	10 (5.8)	0.770
Health professional who taught how to perform CC				
Endocrinologist	3 (1.7)	25 (14.5)	80 (46.2)	0.782
General Practitioner	0 (0)	2 (1.2)	3 (1.7)	0.647
Nutritionist	1 (0.6)	20 (11.6)	66 (38.2)	0.226
Nurse	0 (0)	3 (1.7)	4 (2.3)	0.443
Psychologist	0 (0)	0 (0)	0 (0)	-
Other	3 (1.7)	9 (5.2)	36 (20.8)	0.331
Received encouragement to perform CC from				
Endocrinologist	3 (1.7)	17 (9.8) ^(−)^	101 (58.4) ^(+)^	0.001 ^†^
General Practitioner	0 (0)	3 (1.7)	1 (0.6)	0.050
Nutritionist	2 (1.2)	12 (6.9) ^(−)^	64 (37.0) ^(+)^	0.047 ^†^
Nurse	0 (0)	2 (1.2)	4 (2.3)	0.782
Psychologist	0 (0)	2 (1.2)	5 (2.9)	0.849
Other	0 (0)	6 (3.5)	25 (14.5)	0.382
I do not receive encouragement	3 (1.7) ^(+)^	16 (9.2) ^(+)^	14 (8.1) ^(−)^	0.001 ^†^

* Chi-square. ^†^ statistically significant. Residue Analysis: ^(+)^ the number of cases in this cell is significantly larger than what would be expected if the variables were independent; ^(−)^ the number of cases in this cell is significantly smaller than what would be expected if the variables were independent. CC = Carbohydrate Counting; SUS = Unified Health System.

## Data Availability

The data presented in this study are available on request from the corresponding author. The data are not publicly available due to the university does not have software that makes databases available online.
